# Yellow Fever at Rio

**Published:** 1901-10-26

**Authors:** 


The Hospital

A Journal of

The Medical Sciences and Hospital Administration.

Vol. XXXI.—No. 787.] Edited by Sir Henry Burdett, K.C.B., and Solomon C. Smith, M.D., M.R.C.P. [October 26, 1901.

### YELLOW FEVER AT RIO.

An eminent Brazilian physician, Dr. de Gouvéa, who for nearly 30 years occupied a distinguished position in Rio de Janeiro, both as a practitioner and a teacher of medicine, but who was compelled to leave the country in consequence of the revolution of 1893, has just contributed to *Le Bulletin Médical* of Paris, where he is now residing, a very interesting historical account of the behaviour of yellow fever in and about Rio from the time of its first introduction in 1847 to the present day. His paper opens with a description of the city of Rio itself, which is built upon a bed of sand, resting upon impervious rock, and covered by a layer, of inconsiderable depth, composed of a mixture of alluvial detritus and town refuse. In these conditions, the sand becomes water-logged, and the water rises from it through the soil above, filling any depressions which may exist there, and producing an enormous number of small surface pools which might be designed for the very purpose of furnishing breeding grounds for the *culices*. At no great distance from the bay, the city is surrounded by a semicircle of mountains, rising to considerable elevations; and subsidiary towns, of which Petropolis may be taken as an example, have been built upon successive plateaus. In 1847, when yellow fever was first introduced into Rio, it prevailed extensively in the city itself, and extended to its immediate environs; but it never appeared either at Petropolis or at any other of the hill towns, although between these and Rio itself there was constant communication. This communication, however, was at that time slow and difficult, being conducted entirely by means of mules, and the journey requiring several days for its completion. *Étapes*, or resting houses, were provided on the way, and at these the wayfarers were accustomed to halt at night. It was not unusual for travellers from Rio to Petropolis to sicken with yellow fever on the way; and they would then remain at an *étape* until the disease terminated either in death or in recovery; but the journey was of sufficient length to cover the whole period of incubation, and no case in which the disease first showed itself after arrival at Petropolis was recorded; neither was there any in which it attacked travellers proceeding in the opposite direction. The clothes of sufferers were constantly being carried on to Petropolis after the death or recovery of their owners; and the bedding at the *étapes* was not subjected to any cleansing process by which disease germs would be destroyed. Practically, therefore, there was no infection, in the presence of circumstances eminently calculated to promote its occurrence. The disease disappeared from Rio in 1862, but was again introduced in 1870, and the city has not since been free from it. In the meanwhile, the hill towns have been connected with Rio by railways; so that the journey to Petropolis is now made in two hours, and large numbers of the Rio merchants reside there, and go to and fro to business every day; a mode of living which implies, of course, a considerable amount of daily passage between the two places by many other persons as well. In these altered circumstances, the hill towns have lost their former immunity, and cases of yellow fever occur in them as well as in Rio; but, according to Dr. de Gouvéa, only in the case of persons who have either slept in Rio, or have remained there after sundown. As Professor in the School of Medicine he has for some years endeavoured to impress the bearing of these facts upon his pupils and upon the public, but the current belief in infection has always been too strong for him, and has led not only to the adoption of many useless precautions, but to the neglect of protective measures which might have been successful. Dr. de Gouvéa illustrates his paper by a series of diagrams, showing a definite relation between the prevalence of yellow fever and the temperature; and he shows also that mosquito life is only sustained in full vigour and activity within the same ranges which are favourable to the prevalence of fever. Nothing can be more important than to have the work of the laboratory thus brought to the test of events occurring upon a large scale, and Dr. de Gouvéa's paper will be welcomed by epidemiologists all over the world. It not only, in the most striking manner, confirms the results of the Cuban experiments, but it opens up, even to the inhabitants of temperate climates, a very large vista of possibilities. The *Culex fasciatus*, which has been proved to communicate yellow fever, is of a different genus from the ague-carrying *Anopheles*; and who shall say that our own familiar *Culex pipiens* is entirely free from the evil practices of his (or rather, as the females are the only blood-suckers, of her) relations? How many trivial ailments are there, as well as some severe ones, the exciting causes of which are even now completely unknown to us ; and as to which we are certainly not in a position to exclude the agency of biting insects. The energies of the anti-vaccinists might perhaps be profitably directed towards an effort to destroy certain "domestic pests," and so to render less probable that "introduction of disease into the blood" which they profess to deplore.

